# Safety and Tolerance of Donor-Derived Mesenchymal Stem Cells in Pediatric Living-Donor Liver Transplantation: The MYSTEP1 Study

**DOI:** 10.1155/2017/2352954

**Published:** 2017-06-27

**Authors:** Steffen Hartleif, Michael Schumm, Michaela Döring, Markus Mezger, Peter Lang, Marc H. Dahlke, Joachim Riethmüller, Alfred Königsrainer, Rupert Handgretinger, Silvio Nadalin, Ekkehard Sturm

**Affiliations:** ^1^Pediatric Gastroenterology and Hepatology, University Hospital Tübingen, Hoppe-Seyler-Straße 1, 72076 Tübingen, Germany; ^2^Department of Pediatric Hematology and Oncology, University Hospital Tübingen, Hoppe-Seyler-Straße 1, 72076 Tübingen, Germany; ^3^Department of Surgery, University Medical Center Regensburg, Franz-Josef-Strauß-Allee 11, 93053 Regensburg, Germany; ^4^Department of General, Visceral and Transplant Surgery, University Hospital Tübingen, Hoppe-Seyler-Straße 1, 72076 Tübingen, Germany

## Abstract

**Background:**

Calcineurin inhibitors (CNI) have significantly improved patient and graft survival in pediatric liver transplantation (pLT). However, CNI toxicity leads to significant morbidity. Moreover, CNIs cannot prevent long-term allograft injury. Mesenchymal stem (stromal) cells (MSC) have potent immunomodulatory properties, which may promote allograft tolerance and ameliorate toxicity of high-dose CNI. The MYSTEP1 trial aims to investigate safety and feasibility of donor-derived MSCs in pLT.

**Methods/Design:**

7 to 10 children undergoing living-donor pLT will be included in this open-label, prospective pilot trial. A dose of 1 × 10^6^ MSCs/kg body weight will be given at two time points: first by intraportal infusion intraoperatively and second by intravenous infusion on postoperative day 2. In addition, participants will receive standard immunosuppressive treatment. Our primary objective is to assess the safety of intraportal and intravenous MSC infusion in pLT recipients. Our secondary objective is to evaluate efficacy of MSC treatment as measured by the individual need for immunosuppression and the incidence of biopsy-proven acute rejection. We will perform detailed immune monitoring to investigate immunomodulatory effects.

**Discussion:**

Our study will provide information on the safety of donor-derived MSCs in pediatric living-donor liver transplantation and their effect on immunomodulation and graft survival.

## 1. Background

In recent decades, pediatric liver transplantation has evolved into a state-of-the-art procedure improving prognosis and quality of life for children and adolescents with terminal liver disease. Immunosuppressive pharmacotherapy including calcineurin inhibitors (CNIs) allows the transplantation of solid organ grafts with reasonable patient and graft survival rates [1–3]. However, long-term continuous exposure to immunosuppressive drugs, such as CNIs, mTOR inhibitors, and steroids, carries with it significant clinical side effects. These include renal dysfunction, arterial hypertension, glucose intolerance, posttransplant lymphoproliferative disorder, and opportunistic infections [4, 5]. These side effects account for significant morbidity after liver transplantation [1, 6, 7]. Children are more affected than adults by the chronic toxicity of immunosuppressive medications, in particular by the toxicity of CNIs [8]. In addition, CNIs are not effective in preventing chronic de novo hepatitis in transplanted allografts. This long-term graft injury is associated with a high risk of developing progressive graft fibrosis after 10 years, and up to 25% of patients need retransplantation [9]. Recently, studies have focused on immunological complications after pediatric liver transplantation, such as humoral graft rejection [10] leading to late allograft failure. Therefore, we need novel immunomodulating approaches to limit the risk of immunosuppressive therapy while continuously improving outcome, achieving optimal cognitive and physical development, and maintaining a high quality of life for liver-transplanted children.

### 1.1. Mesenchymal Stem Cells

Mesenchymal stem cells or, according to the terminology of the International Society of Cellular Therapy (ISCT), multipotent mesenchymal stromal cells [11], are adult progenitor cells originating in the neural crest and mesoderm. MSCs can be isolated from the bone marrow and many other sources, including adipose tissue and Wharton's jelly. MSCs can differentiate into mesenchymal cells like osteoblasts, chondrocytes, and adipocytes; in addition, MSCs may potentially differentiate into cell types normally derived from the ectoderm or endoderm, such as hepatocytes [12]. According to ISCT criteria, human MSCs are characterized by their ability to adhere to plastic, their differentiation potential, the presence of stromal cell markers, and the absence of hematopoietic cell markers [11]. MSCs can be used either fresh after culture or after cryopreservation without loss of phenotype or differentiation potential [13], supporting easy clinical application.

Numerous experimental studies imply that MSCs play a role in modulation of immune responses: in mixed lymphocyte cultures, human MSCs have a significant suppressive effect on T-cell proliferation by means of cell-cell interaction and secretion of soluble factors [14]. Mediators secreted by MSCs include, for example, galectin-1 [15]; TGF-*β*1 (transforming growth factor); hepatocyte growth factor [14]; soluble human leucocyte antigen-G5; TNF-stimulated gene 6 protein [16]; and prostaglandin E2. The enzyme indoleamine-2,3-dioxygenase (IDO) is another important factor of immune regulation by MSCs [17]. Together, these factors are able to modulate immune activation of T-cells [18–20], B-cells [21], and macrophages [22], which, in turn, adopt a regulatory phenotype. Furthermore, MSCs affect the differentiation, maturation, and function of dendritic cells [23].

Preclinical models have confirmed these beneficial immunomodulatory effects of MSCs [18, 24, 25]. The first clinical applications have been to treat allo- and autoimmune disorders such as steroid-resistant graft-versus-host disease [26] and Crohn's disease [27]. These applications have been shown to be safe and very promising in terms of clinical efficacy.

### 1.2. MSCs and Solid Organ Transplantation

Mesenchymal stem (stromal) cells (MSCs) may represent an attractive therapeutic option in solid organ transplantation because they modulate immune response and promote regeneration [28]. Systemic application to patients has shown them to be very well tolerated. Clinical studies focusing on the use of autologous [29–31] and allogeneic MSCs [32, 33] in kidney transplantation have been completed, demonstrating safety and feasibility. These studies have shown lower incidence of acute rejection and improving renal function one year after transplantation, illustrating the immunosuppressive properties of MSCs. In adult liver transplantation, the first clinical phase I/II studies of the use of MSCs are ongoing (NCT01841632, NCT01429038, and NCT01844063). Preliminary reports showed that systemic and intraportal infusions of allogeneic MSCs after deceased and living-donor liver transplantation were safe and well tolerated by transplant recipients [34, 35]. None of the previous studies using comparable cell dosage demonstrated any significant side effects of MSC infusion.

Due to the aforementioned toxicity of CNI and insufficient long-term outcome results after liver transplantation, children may benefit from alternative approaches to immunomodulation that will prolong graft survival while reducing CNI toxicity, improving quality of life and promoting long-term allograft tolerance. However, in the context of pediatric living-donor liver transplantation, the safety and feasibility of intraportal and intravenous application of MSCs remain to be proven.

## 2. Methods and Design

### 2.1. Objectives and Endpoints

**Figure 1:**
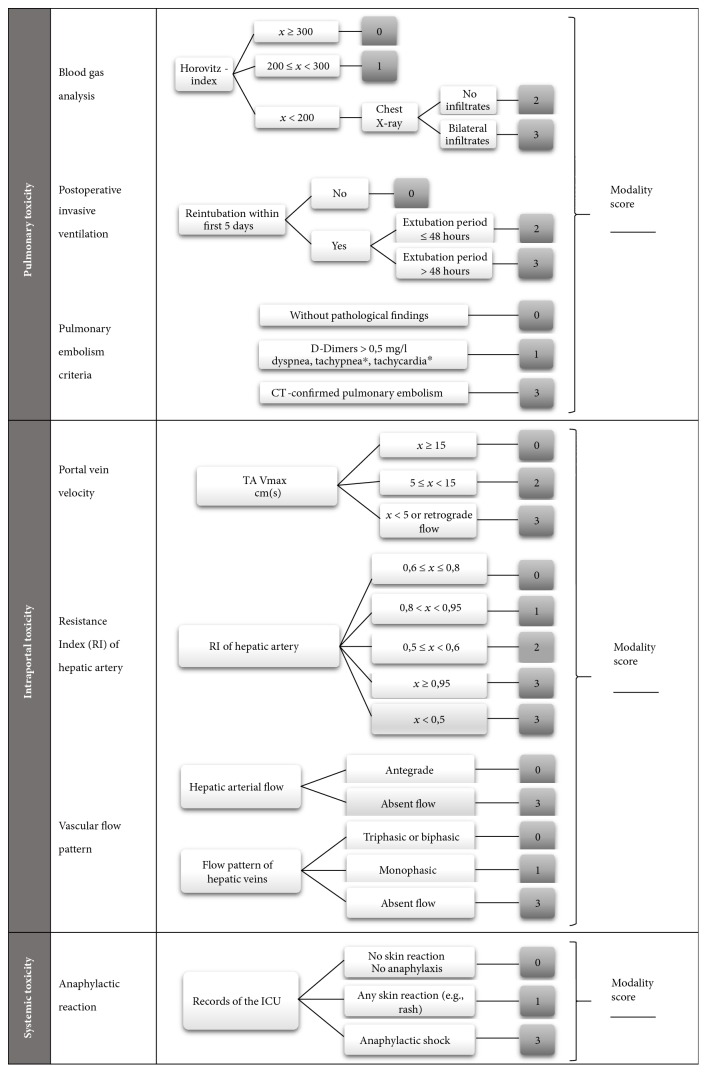
MYSTEP score monitoring infusional toxicity. Cut-off levels are defined upon current publications in adult [61, 62] and pediatric [63, 64] liver transplantation. ^∗^Tachycardia or tachypnea are defined as elevation of age-related range.

The primary objective of this pilot trial is to assess the safety of donor-derived MSC infusions in children undergoing LDLT. Safety will be determined by the following:

1. Incidence, timing, and severity of acute complications related to MSC infusion, using a specific toxicity scoring system (MYSTEP score, Figure 1)
2. Incidence of severe adverse events (SAEs) and their relation to investigational treatment
3. Graft integrity and function after liver transplantation, as measured by aminotransferase and gamma glutamyl transferase activity, bilirubin, albumin, and INR.

**Figure 2:**
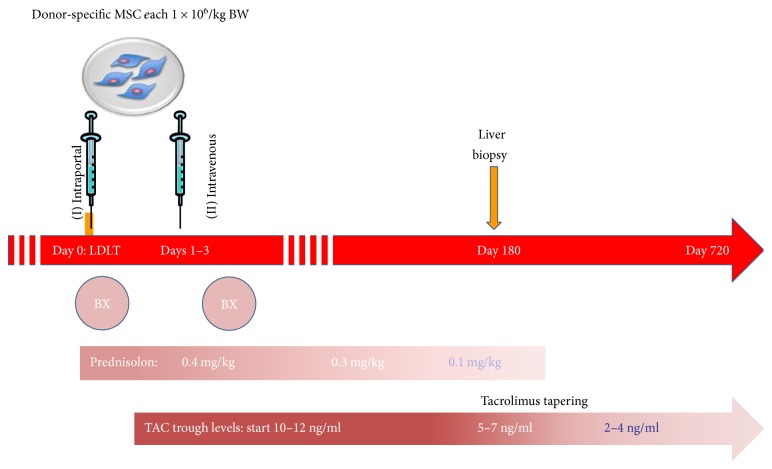
Scheme of investigational treatment and IS tapering strategy. BX = basiliximab; TAC = tacrolimus.

Further, this study aims to evaluate the following:

1. Efficacy
  - Feasibility and safety of tapering immunosuppressive medication according to standard guidelines [36] and according to the step-wise tapering protocol, beginning 6 months after pLT (Figure 2)
  - Time to first biopsy-proven acute rejection (BPAR).
2. Hematologic and immunologic function, as measured by characterization and quantification of mononuclear cell populations, detection of donor-specific antibodies (DSA) and liver autoantibodies, and analysis of a protocol transplant biopsy.
3. Patient and graft survival at 1 and 2 years posttransplantation.

### 2.2. Study Design

The MYSTEP1 (Mesenchymal Stem Cells in Pediatric Liver Transplantation) trial is a 24-month, nonrandomized, open-label, prospective, single-center pilot trial. In total, a minimum of 7 de novo liver recipients, 0–17 years of age, will be recruited at the University Hospital of Tübingen. We will enroll them in the study upon the consent of their legal representatives and upon meeting the eligibility criteria. The study group may be expanded to as many as 10 patients after consulting the data safety monitoring board (DSMB), which will monitor study progress. The board comprises a pediatrician, a transplant surgeon, and a biometrician not otherwise involved in the trial. We have obtained regulatory approval from the Ethical Committee of the University of Tübingen and from the German Federal Institute for Vaccines and Biomedicines (Paul-Ehrlich-Institut; EudraCT number 2014-003561-15). This trial is registered with Clinicaltrials.gov NCT02957552.

### 2.3. Inclusion Criteria

Patients eligible for inclusion in this study must fulfill all of the following criteria:

1. Patient and both parents and/or legal guardian must have given written informed consent.
2. Patients will undergo living-donor liver transplantation for chronic terminal liver failure.
3. Age ≥ 8 weeks and ≤18 years.
4. Body weight > 5 kg.

### 2.4. Exclusion Criteria

Patients fulfilling any of the following criteria are not eligible in this study:

1. Living donor not suitable according to donor and recipient criteria
2. Pregnant or breastfeeding
3. Refusal of adequate contraception (if appropriate)
4. Acute liver failure or highly urgent transplantation
5. Receiving any form of solid organ retransplantation
6. Multi-organ-transplantation
7. Active autoimmune disease
8. Preexisting renal failure with eGFR < 50 ml/min/1.73 m^2^ or require hemodialysis
9. Reduced pulmonary function (lung function test in children older than 6 years: FEV1 and FVC < 70% of age-appropriate norm) or clinical suspicion of pulmonary disease affecting the patient's physical performance, requiring invasive or noninvasive mechanical ventilation
10. History of pulmonary embolism
11. Pulmonary hypertension and/or right ventricular load in echocardiography
12. Reduced cardiac function: left ventricular shortening fraction < 25%
13. Clinically significant systemic infections
14. Undergoing critical care treatment like mechanical ventilation, dialysis, or vasopressor agents
15. Seropositivity for HIV, HTLV, or hepatitis B/C
16. Hepatobiliary malignancies or history of any extrahepatic malignancy
17. Thrombophilia
18. Budd-Chiari syndrome
19. Preexisting thrombosis of portal vein
20. Doppler-sonographic evidence for relevant portosystemic shunts, for example, persistent ductus venosus
21. Cold ischemia time > 90 min
22. Known abuse of drugs or alcohol
23. Known allergy to DMSO.

### 2.5. MSC Culture

Donor-derived mesenchymal stem cells will be obtained 4 weeks before the planned LDLT via bone marrow puncture of the living donor. All donors will undergo routine examination and screening tests including an extensive infectious disease work-up, according to our transplant center's donor screening protocol [37, 38]. Donors must also gain approval from the living donation committee. For MSC preparation, about 20 ml of bone marrow will be taken from the iliac crest under local anesthesia. The processing and expansion of the cells will take place at the Good Manufacturing Practice (GMP) facility of University Children's Hospital, Tübingen. MSCs are cultivated in cell culture flasks using animal-free cell media. For MSC culture, we use human albumin, plasma, and platelets that are obtained from healthy blood donors according to German Blood Transfusion Law. Normally, cultivation takes about 20 days and 2-3 passages are needed. With our culture method, we obtain about 50 × 10^6^ cells with 20 ml bone marrow. Only in rare occasions, more bone marrow is needed for MSC therapy in pediatric recipients.

The cell product can only be released if the following criteria have been fulfilled: regular surface marker expression (CD105+CD73+CD90+CD45-HLA-DR-cells > 90%; CD3+, CD19+, and CD14+ cells < 0.5%); spindle-shaped morphology; a colorless cell suspension; viability of cells of >80%; absence of microbial contamination using culture, mycoplasma PCR, and endotoxin testing; and absence of cell aggregates. The MSC product is cryopreserved with 10% DMSO until designated application (storage at <−150°C in gas phase of liquid nitrogen). On the day of cell transfusion, the assigned dose of mesenchymal stem cells will be thawed at the GMP stem cell laboratory, washed, and suspended in an appropriate volume of isotonic saline with 0.5% albumin at a cell concentration ranging between about 1 and 1.5 × 10^6^ cells/ml.

### 2.6. Study Treatment

The investigational treatment will consist of two transfusions of donor-derived mesenchymal stem cells, each dose ~1 × 10^6^ MSC/kg body weight, with the first infusion intraoperatively (day 0) and the second infusion postoperatively, on days 1–3 after living-donor liver transplantation (Figure 2). Further, all study participants will initially continue on the standard immunosuppressive regimen consisting of basiliximab, corticosteroids, and tacrolimus (tacrolimus trough level 5–10 ng/ml after 6 months), in accordance with our center's pediatric LT protocol. If the protocol liver biopsy 6 months post LT is unremarkable and there is no history of rejection, immunosuppressive medication can be reduced according to the stepwise tapering protocol aiming for a tacrolimus trough level of 2–4 ng/ml (Figure 2). In close collaboration with the responsible regulatory authorities, we have planned a staggered approach: the first three patients will receive study treatments one at a time, with a safety interval of 30 days. Consultation with the DSMB will be required before we resume patient recruitment.

Postoperative venous thrombosis prophylaxis is mandatory using low-dose heparin. After about 10 days, prophylaxis should be switched to aspirin and maintained for 3 months after LDLT. Prophylaxis against bacterial, fungal, and viral infections will adhere to our center's pLT protocol.

### 2.7. Data Collection

Children enrolled in this study will undergo a standard pretransplant work-up, which consists of baseline clinical data (demographics, medical history, current medication, physical examination, laboratory examinations, thrombophilia screening test, urinalysis, electrocardiogram, abdominal ultrasound, and chest X-ray). Pregnancy tests will be performed using a test for *β*-hCG in serum on all female participants with childbearing potential (those age 9 years or older) during the screening visit.

### 2.8. MSC Infusion

The first administration of 1 × 10^6^ cells/kg body weight will be performed intraoperatively via portal infusion after complete liver allograft reperfusion (day 0). MSC suspension will be administered via a small venous catheter into the portal vein for 20 minutes while gently waving the syringe to keep cells in suspension. In addition to using Doppler ultrasonography, we will measure portal flow by transit time flow measurement (Medistim®) during cell infusion [39, 40]. Cell infusion should be discontinued if the portal flow decreases significantly (below 20 ml/min/100 g liver weight). The second MSC administration, dosing 1 × 10^6^ cells/kg body weight, will be performed via a systemic intravenous route on postoperative day 2 (time window ± 1 day). MSC suspension will be transfused via a central venous catheter or peripheral venous catheter for 20 minutes under sterile conditions. During cell infusions, we will continuously monitor the patient's pulse, blood pressure, oxygen saturation, respiratory rate, and body temperature.

### 2.9. Follow-Up Visits

We will see patients frequently for follow-up visits during the first 28 days after transplantation. Study visits will consist of regular clinical examinations, Doppler sonography, and blood tests aimed at early detection of treatment-emergent events. We will assess the patient's MYSTEP toxicity score on days 0, 2, 4, 7, 10, and 28 after LDLT. Additional study visits will be performed up to 720 days after LDLT to assess allograft survival, incidence of rejection, incidence of (opportunistic) infections, kidney function, and individual need for immunosuppressive medication (Table 1). If the patient follows an unremarkable clinical course and a normal protocol liver biopsy, immunosuppressive drugs will be gradually reduced after six months (Figure 2), aiming at tacrolimus trough levels of 4 ng/ml or below after 12 months. After termination of the study, participants will be followed in our outpatient clinic for another five years. We will monitor the patients for long-term allograft function, extrahepatic organ function, and long-term complications, particularly the occurrence of malignancy.

#### 2.9.1. MYSTEP Score

In order to evaluate and quantifiy treatment-emergent adverse events of MSC infusion, we defined a pediatric infusional toxicity score that adopts the MiSOT-I score for adults [41]. The score focuses on three independent modalities reflecting injury to the lungs and to the liver allograft, for example, by thrombembolism, and systemic reactions, such as anaphylaxis (Figure 1). For each of these three modalities, degrees of severity between 0 (no treatment-emergent adverse event) and 3 (severe treatment-emergent adverse event) were defined. Clinical data, blood gas analysis, chest X-ray, and doppler-ultrasound will be obtained on designated study visits (Table 1). The occurrence of two consecutive grade 3 events will be reported as severe adverse event. The MYSTEP score was validated retrospectively by analysing our cohort of pediatric LT recipients without investigational treatment [42].

#### 2.9.2. Protocol Liver Biopsy

In this study, we will perform a protocol liver biopsy 6 months after LDLT and MSC infusion. A percutaneous liver biopsy will be performed under sonographic control and in analgosedation, according to recommendations of the ESPGHAN Hepatology Committee [43] and local standards. Additionally, we will perform protocol liver biopsies routinely every 5 years after LT. Liver tissue will be processed for immunohistochemistry (hematoxylin and eosin staining; staining for CD3, CD4, and CD20). Biopsies will be scored according to the Banff criteria and the liver allograft fibrosis scoring system [44]. Further, expression of anti- and proinflammatory cytokines will be measured by real-time RT-PCR in liver tissue.

#### 2.9.3. Immune Monitoring

Additional blood samples will be collected to investigate surrogate markers of the participant's immune response status (Table 1). This immunological monitoring will include lymphocyte proliferation assay to evaluate antidonor reactivity, flow cytometry to describe the recipients' leucocyte phenotypes and presence of donor leucocytes (HLA chimerism), and serum analysis to screen for DSA, liver-directed autoantibodies, and inflammatory cytokines. Further, we will screen for serological markers of iron hemostasis, that is, ferritin and hepcidin, which presumably play a role in development of operational tolerance after liver transplantation [45].

### 2.10. Risk-Benefit Assessment

MSCs may support induction of allograft tolerance and help to achieve long-term tolerance [25]. Patients may need smaller amounts of immunosuppressive drugs, which are associated with a risk of toxicity and in many cases prove fail to prevent long-term damage of the allograft. In addition, MSCs have the potential to foster regeneration of transplanted organs, for example, following ischemia-reperfusion injury [46, 47]. The associated risks of MSC therapy in pediatric LT recipients are unknown. For indications other than solid organ transplantation, the systemic application of MSCs in children has been shown to be safe, and no treatment-emergent adverse events have been reported [26, 48]. However, potential risks include transmission of infectious disease, thromboembolism, portal vein thrombosis, anaphylaxis, and carcinogenic effects [49]. These risks require preventive measures and continuous monitoring. In our own experience, more than 100 applications of MSCs in children in the setting of treatment for GvHD and Crohn's disease have shown MSCs to be well tolerated without occurrence of severe adverse events. Further, infusion of donor-derived MSCs bear a theoretical risk of sensitization by donor antigens, which could lead to formation of de novo DSAs and rejection. However, clinical studies using donor-derived MSCs after kidney transplantation [32, 33] demonstrated fewer rejection episodes and better allograft function one year after transplantation. On the basis of current experience, we believe that the potential for beneficial effects of MSC administration after liver transplantation and the limited potential risks of adverse side effects justify participation in this study.

## 3. Discussion

Introduction of current standard immunosuppressive therapies including CNIs has had a major impact on reduction of acute mortality after pediatric liver transplantation [2, 50]. However, life-long exposure to chronic pharmacological immunosuppressants impairs quality of life and reduces long-term survival. Children are particularly affected by chronic drug toxicity and long-term allograft failure [5]. These limitations motivate our search for alternative cellular treatment strategies for achieving allograft tolerance after pediatric liver transplantation. The immunomodulatory properties of mesenchymal stem cells, tested in both in vitro and in vivo models and in early clinical trials, may make them suitable for improved immunomodulation in pLT. The addition of MSCs to current immunosuppressive strategies can help to reduce the level of toxic CNIs and limit ischemia/reperfusion injury [47] while improving graft survival. Preclinical data suggest that MSCs may contribute to long-term allograft tolerance by induction of tolerogenic regulatory T-cells [18–20] and macrophages [22]. The first clinical studies of kidney transplantation have demonstrated the safety of MSC infusion and indicated the efficacy of MSCs in reducing allograft rejection and interstitial fibrosis [30, 31]. Further, clinical studies of the efficacy of allogeneic MSCs in liver transplantation are pending. Preliminary results underline a beneficial safety profile upon clinical application of this cell type [34, 35].

To our knowledge, this is the first clinical trial of immunomodulating therapy with mesenchymal stem cells in pediatric solid organ transplantation. In this pilot study, we aim primarily to determine safety and feasibility of intraportal and intravenous infusion of donor-derived MSC in children undergoing LDLT. We will assess safety based on incidence of acute infusion-related complications measured by the MYSTEP score, on occurrence of severe adverse events and on allograft function after pLT. We have designed our study protocol to optimize prevention of adverse events and to identify any that arise early. For example, using a “staggered approach” to patient recruitment and ensuring intraoperative quantitative monitoring of graft perfusion by transit time flow measurement will help ensure a very high level of safety for the children who participate. In adults, no toxicity has been observed during intravenous and intraportal infusion to date [51].

Previous studies of MSCs in kidney transplantation have shown contradictory data on the risk of overimmunosuppression, which may lead to opportunistic infections [30, 31]. This safety issue might play an important role in pediatric liver recipients, since they are more frequently naïve for EBV or HCMV infections compared to adult LT recipients. Therefore, as part of follow-up after pLT and MSC infusion, it is essential to frequently and accurately monitor infectious complications and TAC trough levels.

Many details of the mechanisms of immunoregulation by MSCs in transplant recipients remain unknown. It is evident, however, that an increase in the percentage of Foxp3 positive regulatory T-cells is one important mode of MSC action in transplant patients [28, 29, 35, 52]. Other cell types which are supposed to mediate the immunomodulatory and regenerative effects are dendritic cells, monocytes, macrophages [22], and MDSCs [20]. In the MYSTEP1 study, immune monitoring will therefore focus on quantitative analysis of leukocyte subpopulations using validated protocols [35, 53]. We will also analyze, in liver tissue, RNA expression of pro- and anti-inflammatory cytokines mediating immunomodulatory effects. Further, eventual humoral alloreactive responses will be monitored by detecting HLA and liver-specific antibodies.

The optimal donor source of MSCs is still unclear. In this trial, we will use bone marrow-derived MSCs obtained from the solid-organ donor. Donor-derived MSCs may contribute to donor-derived allograft tolerance [54]. Living-donor liver transplantation is a standard procedure in children and offers the opportunity to obtain MSCs and graft tissue from an identical living donor, frequently a parent. However, sensitization of the recipient and formation of de novo DSAs may constitute an adverse effect of allogeneic MSCs. A study of renal transplantation in an animal model reported increased allograft rejection and an increase of DSAs when donor-derived MSCs were administered 4 days before transplantation [55]. This supports the hypothesis that timing of infusions and initial concomitant immunosuppressive treatment are crucial [56]. In this regard, previous clinical studies administering allogeneic, donor-derived MSCs at the time of kidney transplantation [32, 33] showed fewer rejection episodes and better allograft function one year after transplantation. In the MYSTEP1 trial, we will regularly screen for the occurrence of de novo DSA.

In addition to the source of MSCs, the time and route of cell application may influence the effectiveness of MSCs. Direct infusion of the cells into the graft can make use of the tissue-repair capacity of MSCs to treat ischemia reperfusion injury. Furthermore, preclinical models showed that in addition to systemic effects, local mechanisms were responsible for transplant tolerance by MSCs [19]. One important mechanism was attenuation of allostimulatory dendritic cells [23]. After intravenous application, the largest fraction of MSCs were pooled in the lungs [57] and lymph nodes and did not reach the liver. Still, most studies in solid organ transplantation apply intravenous MSC infusion. The observed immunomodulatory effects may be mediated longer term by other host cells [58]. Based on these considerations, in the MYSTEP1 trial, we will administer the first dose of MSCs intraportally at the end of the LDLT procedure and administer the second dose intravenously on postoperative day two [49].

In keeping with our focus on safety and feasibility, all participants will be treated in combination with the center's standard immunosuppressive regime, which consists of basiliximab, tacrolimus, and steroids. Preclinical studies suggested that CNIs and glucocorticoids may affect MSC morphology, migration, and immunomodulatory behavior, possibly affecting the success of the cell therapy [59, 60]. However, current studies of adult kidney transplant recipients that have applied MSCs in combination with steroids and CNIs [29–33] have demonstrated potential effectiveness in spite of the concomitant use of CNIs and steroids. To address questions about the synergistic or counteractive effects of immunosuppressive medication on MSC function, a planned phase 2 clinical study will focus on the optimization of an immunosuppressive treatment regime in combination with MSC infusions.

In conclusion, MSCs have the potential to become part of an array of novel treatment options for pediatric LDLT recipients aimed at promoting allograft tolerance and improving long-term allograft survival while reducing toxicity of chronic IS treatment. A positive outcome of the MYSTEP1 trial in terms of safety and allograft survival would constitute a major advancement in pediatric solid organ transplantation. Subsequently, we intend to conduct a second, larger multicenter trial to study the immunomodulatory efficacy of MSC treatment protocols for improving long-term allograft tolerance in pediatric liver transplant recipients.

## Figures and Tables

**Table 1:** Assessment schedule MYSTEP1 study.

Visit	BL	MSC1	MSC2	Follow-up visits									
Days after LT	−28	0	2 ± 1	4	7	10	28	90	180	270	360	540	720
Informed consent Inclusion and exclusion criteria	x												
Concomitant medication			x	x	x	x	x	x	x	x	x	x	x
TAC dosage			x	x	x	x	x	x	x	x	x	x	x
Anthropometric parameters	x	x					x	x	x	x	x	x	x
Vital signs	x	x	x	x	x	x	x	x	x	x	x	x	x
Physical examination	x	x	x	x	x	x	x	x	x	x	x	x	x
Tacrolimus blood trough level			x	x	x	x	x	x	x	x	x	x	x
Routine laboratory including liver parameters	x	x	x	x	x	x	x	x	x	x	x	x	x
eGFR	x				x		x	x	x	x	x	x	x
Virus PCR: EBV, HCMV, and ADV	x				x		x	x	x	x	x	x	x
HHV-6	x				x		x						
Doppler ultrasonography	x	x	x	x	x	x	x	x	x	x	x	x	x
Infusional toxicity score		x	x	x	x	x	x						
TNF-*α* and IL-6 serum levels	x		x	x	x								
Immune monitoring	x				x		x		x		x		x
Antibodies: DSA; ANA, SMA, and LKMA	x								x		x		x
Percutaneous liver biopsy									x				
MSC administration		x	x										

BL: baseline; MSC1: first intraoperative MSC infusion; MSC2: second MSC infusion on postoperative day 2.

## Abbreviations

ADV: Adenovirus
ANA: Antinuclear antibody
BPAR: Biopsy-proven acute rejection
DSA: Donor-specific HLA-antibody
HCMV: Human cytomegalovirus
DMSO: Dimethylsulfoxide
DSMB: Data safety monitoring board
EBV: Epstein-Barr virus
INR: International normalized ratio
IS: Immunosuppression
ICU: Intensive care unit
ISCT: International Society of Cellular Therapy
LDLT: Living-donor liver transplantation
LKMA: Anti-liver-kidney microsomal antibody
LT: Liver transplantation
MDSC: Myeloid-derived immunosuppressive cell
MSC: Mesenchymal stem (stromal) cell
pLT: Pediatric liver transplantation
RI: Resistance index
RT-PCR: Reverse transcription polymerase chain reaction
TA Vmax: Time-averaged maximum velocity
SAE: Severe adverse event
SMA: Anti-smooth muscle antibody.
